# Notifications of work-related injuries and diseases: an observational study on a mining disaster

**DOI:** 10.1186/s12913-023-09838-1

**Published:** 2023-08-31

**Authors:** Rita de Cássia Costa da Silva, Maykon Anderson Pires de Novais, Paola Zucchi

**Affiliations:** 1 Graduate Program in Translational Medicine, Department of Medicine, Escola Paulista de Medicina, Federal University of São Paulo, São Paulo, Brazil; 2 Discipline of Health Economics and Management, Department of Medicine, Escola Paulista de Medicina, Federal University of São Paulo, São Paulo, Brazil

**Keywords:** Health Services Management, Occupational Health, Notification of Diseases, Unified Health System, Health Effects of disasters, Occupational Diseases

## Abstract

**Introduction:**

Accidents at work are events that endanger the health and life of workers. They are considered a public health problem, being the object of studies and actions in the Brazilian health system. The collapsed of the ore tailings dam in the municipality of Brumadinho, Brazil, in January 2019, shocked the world due to the proportion of environmental and human damage caused. In this context, concern for the health of workers gained prominence. This paper evaluated the impact of the collapse of the mining tailings dam in Brumadinho, on notifications of occupational health problems before and after the disaster.

**Methods:**

An observational, longitudinal and retrospective study, of the time series type with a statistical approach was carried out on notifications of work-related injuries and diseases, available in the local database of the Notifiable Diseases Information System between January 2017 and December 2021.

**Results:**

During the study period, 520 notifications of work-related injuries and conditions were registered. Of this total, 67.3% were serious work accidents, 26.0% mental disorders, 12.3% accidents with biological material and 2.9% repetitive strain injuries/musculoskeletal disorders, occupational dermatoses and induced hearing loss by noise. Occurrences were more frequent in 2019, the year in which the mining disaster occurred, recording 65.2% of total notifications. Regarding the volume of notifications after the disaster, there was a statistically significant reduction (p < 0.001) for accidents with biological material; significant increase for severe work accident and mental disorder. The average time between the occurrence of the injury and the notification showed a statistically significant reduction for accidents involving biological material (p = 0.001) and a significant increase for serious accidents at work (p = 0.016).

**Conclusions:**

It was demonstrated that there were changes in the number of notifications when comparing the period before and after the mining disaster, with a consequent impact on the health of workers, which may persist over the years.

Accidents at work are defined as events that endanger the life and health of formal workers. In Brazil, Law 8.213/91 characterizes an accident at work as one that occurs due to the exercise of work at the service of the company, causing bodily injury or functional disturbance that causes death or permanent or temporary loss or reduction of the ability to work. Likewise, occupational diseases are understood as those produced by professional practice or triggered by the conditions to which the worker is exposed during the exercise of their professional activities [1].

Occupational accidents, as well as occupational diseases, are a serious public health problem, being the subject of studies and actions in the Unified Health System, through Occupational Health Surveillance. Worker health care is part of the field of public health management and cannot be disconnected from the care provided to the general population [2]. However, from the perspective of this study, aspects aimed at coping with conditions that pose a risk to the health of workers are highlighted, both in the sense of providing information on preventive measures, and in mitigating the effects and impacts of new disasters.

On January 25, 2019, at 12:28 pm, the tailings dam at the Córrego do Feijão mine collapsed, shocking the world due to the resulting environmental and human damage. Around 11.7 million cubic meters of tailings spread across the region, reaching the Paraopeba River and compromising the water supply to several surrounding cities. The mine was owned by the mining company Vale, a Brazilian multinational located in the municipality of Brumadinho in the metropolitan region of Belo Horizonte in the state of Minas Gerais, Brazil [3].

This serious disaster altered the daily life of the local population, with impacts on health, especially of the workers directly involved in the operations related to the event. Official data provided by the mining company indicates that there were 270 deaths, while the Association of Relatives of Victims and Affected by the Failure of the Córrego do Feijão Mina Dam (AVABRUM) maintains that there were 272 fatalities due to the inclusion of two unborn children [4].

The disaster had a direct impact on the health conditions of the population, with both immediate effects and effects that may persist for many years [5]. In Brazil, the Unified Health System must guarantee universal, full and equitable access of the population to health actions and services through public policies [6]. In view of this, public health managers and planners need to have access to data and scientifically based evidence to guide decision-making, prioritizing investments to meet the needs of the affected populations.

In the Brazilian health system, the surveillance of occupational health risks is performed through the Notifiable Diseases Information System (SINAN), which contains information from notifications and investigations of notifiable diseases, described in Consolidation Ordinance No. 4 of September 28, 2017, Annex V – Chapter I, and the Sentinel Network for Compulsory Notification of Work-Related Accidents and Illnesses, established by the Brazilian Ministry of Health, in article 2 of Ordinance GM/777, of 2004, for the rapid identification, investigation and notification of work-related diseases. The use of SINAN is decentralized, which promotes the generation of a substantial amount of epidemiological information for the Brazilian territories and which assists in locoregional planning. Despite the limitations of SINAN, as shown in previous studies, the system is the official repository for data on notifications of occupational health problems [7]. In this sense, the analysis of this database can contribute to the improvement of health management, as it demonstrates the effects of work conditions and processes on the health of the working population.

There are global records of various natural or man-made disasters that have affected the health of populations. After the nuclear accident in Fukushima, in Japan, which occurred in 2011, scholars identified immediate impacts on the mental health of workers [8]. They pointed out the need to develop studies to monitor the long-term effects of accidents that compromised the health of workers [9]. Global weather events have been the subject of concern, due to the increase in frequency and intensity, and the effects they produce on workers’ health [10].

A study carried out in 2018, in Brazil, showed that the number of accidents at work and the number of deaths resulting from them is high [11]. However, studies that analyze the effects of mining disasters on workers’ health are still rare in Brazil, despite the volume of data on notifiable diseases and injuries available in the Notifiable Diseases Information System (SINAN) [12].

In this context, this study sought to analyze SINAN notifications to characterize the injuries and diseases related to the health of workers before and after the collapse of the mining tailings dam in the municipality of Brumadinho to identify the impact of the dam collapse on the health of workers.

## Methods

A retrospective, longitudinal and observational study, of the time series type with a statistical approach was conducted based on the analysis of secondary data for the period of 2017–2021. The study examined data from a two-year interval before the disaster (January 25, 2017 (T -2) to January 24, 2019 (T -1)), the year of the disaster (January 25, 2019 to January 24, 2020 (T 0)), and two years after the mining disaster (January 25, 2020 (T 1) to January 25, 2021 (T 2)), which occurred in Brumadinho, Minas Gerais, Brazil. The anonymized data were provided by the Municipal Health Department, upon signature of a Data Use Commitment Term.

All notifications containing data on diseases and work-related injuries available in the local SINAN database and provided by the Brumadinho Municipal Health Department were eligible for inclusion in the study. Notifications not related to work or that contained inconsistent information were excluded from the study. Individually identifiable data were anonymized to comply with the ethical criteria for the study.

The database was organized in an electronic spreadsheet, and a preliminary query was performed on the SINAN database to determine the volume of notifications and their consistency. To compare the findings, injuries that occurred on January 25, 2019, the day on which the disaster occurred, were classified as occurring “after” the disaster. The data were tabulated by year to characterize the period before (T -2 to T -1) and after (T 0 to T 2) with T0 being the year of the disaster.

The study variables were obtained from the notification forms of work-related injuries and diseases diseases described in the National List of Compulsory Notification of Public Health Diseases, Conditions and Events [13] and in the national list of diseases monitored by the sentinel surveillance strategy for occupational health [14].

To identify the statistical significance in the volume of notifications before and after the disaster, the chi-square test was used. The T Student and Anova tests were used to verify the significance of the variables between the time of occurrence of the injury and its notification and Tukey to identify the relationship between the variable time and the number of notifications.

This study was approved by the Research Ethics Committee of the proposing institution.

## Results

In the period from two years before to two years after the disaster (T -2 to T 2), 520 notifications of work-related health injuries and diseases were identified, representing 1.5% of the municipality’s population, considering the last count available, which informed 33,973 people (IBGE, 2010) and 4.2% of the employed population (12,330 people).

The injuries and diseases notified during the study period and the respective ICD-10 (International Disease Code) were Accident with biological material (Z20.9); Serious Work Accident (Y96); Dermatoses (L98.9); Repetitive Strain Injury/Work Related Musculoskeletal Disorders (Z57.9); Noise-Induced Hearing Loss (H83.3) and Mental Disorders (F99).

The most notifications during the period T-2 to T + 2 reported serious workplace accidents (Y96), with a total of 350 (67.3%) cases, followed by mental disorders (F99), with 91 (17.5%) cases, and accidents involving biological material (Z20.9), with 64 (12.3%) cases. There were a total of 15 (2.9%) notifications of other diseases or conditions, such as repetitive strain injury/musculoskeletal disorders, occupational dermatoses and noise-induced hearing loss, during the observed period. There were no reports of work-related cancer (C80) or pneumoconiosis (J64) during the study period.

Notifications were the most frequent in 2019, year of disaster occurrence, (T 0), with 339 (65.2%) of the total notifications registered in this period (Table 1).

**Table 1:** Reported work-related injuries and diseases, Brumadinho (Brazil), 2017–2021 (T -2 to T 2)

Injury (ICD)		T -2	T -1	T 0	T 1	T 2	Total
Serious accident at work (Y96)	N	11	7	265	54	13	**350**
	%	3,2	2,0	75,7	15,4	3,7	**100,0**
Mental disorder (F99)	N	0	1	55	12	23	**91**
	%	0,0	1,1	60,4	13,2	25,3	**100,0**
Accident involving biological material (Z20.9)	N	16	22	14	10	2	**64**
	%	25,0	34,4	21,9	15,6	3,1	**100,0**
Repetitive strain injury/work-related musculoskeletal disorder (Z57.9)	N	2	1	5	4	0	**12**
	%	16,7	8,3	41,7	33,3	0,0	**100,0**
Dermatosis (L98.9)	N	0	0	0	2	0	**2**
	%	0,0	0,0	0,0	100,0	0,0	**100,0**
Noise-induced hearing loss (H83.3)	N	0	0	0	1	0	**1**
	%	0,0	0,0	0,0	100,0	0,0	**100,0**
**Total**	N	**29**	**31**	**339**	**83**	**38**	**520**
	%	**5,7**	**5,9**	**65,2**	**15,9**	**7,3**	**100,0**

Source: Research data

Although the largest number of notifications occurred after the disaster, for the 64 cases of accidents involving biological material, it was observed that 40 (62.5%) notifications occurred before the disaster (T-2 a T-1) and that 24 (37.5%) occurred after (T0 a T2). The chi-square test showed a statistically significant reduction (*P* < 0,001) for these cases.

For serious workplace accidents, 350 cases were identified, with 22 (6.3%) notifications occurring before the disaster and 328 (93.7%) occurring after. Notably, the largest number of occurrences occurred on the day of the disaster, which is considered to be “after” the disaster here for the purposes of statistical analysis of the chi-square test indicated in a significant increase (*P* < 0,001) of these cases.

In the case of mental disorders, only 2 (2.2%) cases were reported in the previous period, and 89 (97.8%) were reported after the disaster of the chi-square test indicated a statistically an significant increase (*P* = 0,001); that is, the increase in the number of cases of mental disorders after the disaster was proportionally greater than that for other diseases related to the health of workers.

Among the other diseases and conditions (repetitive stress injury/work-related musculoskeletal disease, dermatoses, noise-induced hearing loss), only 5 (33.3%) notifications were identified before the disaster, while 10 (66.7%) were identified after. The result of the chi-square test (p = 0,020) showed that the increase in the number of notifications after the disaster was proportionally lower than that for the other diseases (Table 2).

**Table 2:** Work-related injuries and diseases reported before (T-2 to T-1) and after (T0 to T2) the mining disaster, Brumadinho (Brazil), 2017–2021

Injury (ICD)		Notifications		Total	p-value
		**T -2 to T -1**	**T 0 to T 2**		
Accident involving biological material (Z20.9)	N	40	24	**64**	**< 0,001**
	%	62,5	37,5	**100,0**	
Serious accident at work (Y96)	N	22	328	**350**	**< 0,001**
	%	6,3	93,7	**100,00**	
Mental disorder (F99)	N	2	89	**91**	**= 0,001**
	%	2,2	97,8	**100,00**	
Repetitive strain injury/work-related musculoskeletal disorder (Z57.9), dermatosis (L98.9), noise-induced hearing loss (H83.3)	N	5	10	**15**	**= 0,020**
	%	33,3	66,7	**100,00**	
**Total**	N	69	451	**520**	
	%	13,3	86,7	**100**	

Source: Research data

Excluding serious work accidents that occurred on the exact day of the disaster, there was still an increase in notifications of these accidents, from 23 (44.2%) in the period before the event to 29 (55.8%) after the event. However, the chi-square test (*P* = 0,037) showed that the increase in serious occupational accidents after the disaster was proportionally lower than that of the other diseases (Table 3).

**Table 3:** Serious Work Accident and other work-related injuries and diseases, excluding cases that occurred on the day of the disaster, Brumadinho (Brazil), 2017–2021

		Notifications		Total	p-value
		**T -2 to T -1**	T 1 to T 2		
Serious accident at work (Y96)	N	23	29	52	= 0,037
	%	44,23	55,77	100,00	
Other health problems	N	48	119	167	
	%	28,74	71,26	100,00	
Total	N	71	148	219	
	%	32,42	67,58	100,00	

Source: Research data

The mean time between an accident involving biological material and the registration of the notification was 62 days for injuries that occurred before the disaster and 10 days for injuries that occurred after the disaster. The T test indicated a statistically significant reduction (*P* = 0,001) in the number of notifications between the periods before and after the disaster. In the case of a serious work accident, the average time between the accident and the registration of the notification was 52 days for injuries that occurred before the disaster, 148 days for injuries that occurred on the day of the disaster and 78 days for those that occurred after the disaster. The ANOVA test resulted in (*P* = 0,016), indicating a statistically significant difference. By Tukey’s method of multiple comparisons, it is observed that the increase in time occurred mainly for cases that occurred on the day of the disaster. In cases of mental disorders, the mean time between diagnosis and notification was 90 days before the disaster, while after the disaster, the mean time increased to 289 days. This increase was not statistically significant (*P* = 0,064), but notably, there was a low number of reported mental disorders before the disaster, with only two cases reported, and there was high variability in the days between diagnosis and notification after the disaster, from zero to 724 days (Table 4).

**Table 4:** Time between occurrence and notification, before (T-2 to T-1) and after (T0 to T2) the disaster, Brumadinho (Brazil), 2017–2021

Parameter	Occurrence of the disease	Accident Involving Biological Material						
		**N**	**Mean**	**Standard deviation**	**Minimum**	**Median**	**Maximum**	**p-value**
**Time (days) between** ** grievance and notification**	T -2 to T -1	40	61,5	78,8	0,0	13,5	295,0	= 0,001
	T 0	0	0	0	0	0	0	
	T 0 to T 2	24	10,3	38,4	0,0	0,0	185,0	
		**Serious Occupational Accident**						
		**N**	**Mean**	**Standard deviation**	**Minimum**	**Median**	**Maximum**	**p-value**
	T -2 to T -1	23	51,5	99,1	0,0	22,0	466,0	= 0,016
	T 0	298	148,0	202,5	0,0	67,0	724,0	
	T 1 to T 2	29	77,9	91,8	1,0	32,0	259,0	
		**Mental Disorder**						
		**N**	**Mean**	**Standard deviation**	**Minimum**	**Median**	**Maximum**	**p-value**
	T -2 to T -1	2	90,5	58,7	49,0	90,5	132,0	= 0,064
	T 0	0	0	0	0	0	0	
	T 0 to T 2	89	289,7	309,2	0,0	148,0	724,0	

Source: Research data

## Discussion

In 2019, the collapse of Vale’s tailings dam increased the number of work accidents in Brazil, with direct and indirect impacts on the health of workers and the resident population. The disaster also caused immediate impacts on the management of the public health system. There has been increasing evidence on the organization and planning of health services in the affected territory, allowing us to understand how managers and health professionals articulated the rapid response to deal with the effects of the disaster. On the other hand, it has become important to generate evidence on the characterization and volume of diseases that affect the population in order to enable actions and responses in the short, medium and long term [15].

A study conducted in Brazil in 2020 using SINAN data corroborates the findings of this study by showing the increase in serious accidents at work. Serious accidents at work were the most registered injury in the notifications available on SINAN, in the period of two years before and two years after the mining disaster. This injury is characterized by the causal relationship with the work activity, resulting in functional impairment, mutilation or death [16]. In addition to the direct impact on the health of the worker, the repercussion in the health system bears high costs related to accidents at work, making them a public health problem [17].

In Brazil, accidents at work occur more frequently in the industrial, agricultural and service sectors than in other sectors [11]. This evidence reinforces the importance of characterizing diseases and conditions related to the health of workers to identify their impact on the health of workers and, consequently, on the public health system. In this sense, this study shows that the numbers of occurrences and notifications were higher after the disaster than before the disaster, which overloaded the health system, considering that outpatient and hospital health units had to pay for the care of workers and the population in the affected areas.

The results showed that occupational health surveillance should be a priority in the health care network to support health promotion and prevention actions together with companies located in the municipality. A recent study in this area showed worrying values for mortality due to accidents at work in Brazil in all regions, even with underreporting in SINAN [16]. This study indicates that the occurrence of serious accidents at work has increased, which is indicative of the need for monitoring and intersectoral evaluation of working conditions.

Mental disorders were the second most frequent condition after the mining disaster in Brumadinho. This study showed a significant increase in mental disorders among workers, which highlights the impact of mining disasters on the mental health of workers. A study conducted in 2019, shortly after the disaster, found that the impact on the mental health of those affected was a consequence of the magnitude of the event combined with the large number of deaths, environmental destruction and socioeconomic consequences, as the municipal economy is dependent on mineral extraction. In addition to the acute reactions identified immediately after the disaster, the authors warned of the risk of the onset of posttraumatic stress in the medium term. They also provided evidence suggesting that the surviving workers could develop mental disorders in the medium and long term due to their exposure to losses and their need to maintain their jobs and income [17].

Mental illness is identified as one of the most common occupational diseases among workers in general. In addition, work-related mental disorders are associated with stressful situations caused by the production process [18]. This situation is worrying, especially because in Brazil, morbidity due to mental disorders is considered high and is associated with other comorbidities such as diabetes and cardiovascular problems. After the episode that occurred in Brumadinho, and based on studies carried out in countries that have already suffered catastrophes, researchers warned of the association between the mining disaster and the occurrence of mental disorders, increased consumption of alcohol, tobacco and other drugs, addition to other chronic noncommunicable diseases in the general population. The great disasters bring as consequences, in the short term, the appearance of conditions compatible with communicable diseases, such as diarrhea, and with the passage of time non-communicable diseases gain evidence [19].

Among workers, there is a greater risk of mental disorder among those exposed to situations with a greater degree of danger [18]. After the dam failure, which caused the death of several workers, the increase in notifications of mental disorders may have been associated not only with the human impact of the disaster but also with the stressful situation of continuing to be employed in mining given the circumstances of the event. Although the findings of this study show an increase in mental disorders among workers, it should be taken into account that the situation may be even more worrying, as previous studies have highlighted the existence of the underreporting of this condition [20].

Accidents involving biological material were identified as the third most frequent problem related to mining disasters. Worldwide, such accidents occur repeatedly among health professionals, especially in the nursing field, due to the characteristics of their work. In general, the inadequate sharps disposal is the main reason for this type of accident. Precautionary actions, such as the use of protective equipment and proper handling, are effective in preventing accidents with biological material [21]. In this study, we did not investigate the causes of accidents involving biological material but rather the frequency. The increase in notifications may have been related to the workload of health professionals as a result of dam failure. Therefore, as already discussed by other authors, risk management is emphasized as part of routine health services and a mechanism for protecting workers [22].

The time elapsed between the occurrence and notification of a work-related illness or accident can be influenced by several factors, depending on the illness or accident. In this study, after the disaster, a long period of time was identified between the occurrence and the notification for cases of serious work accidents and mental disorders. Although this study did not investigate the factors that could determine the length of time between the occurrence and the notification, it should be noted that the delay may be a factor indicative of the fragility of the occupational health surveillance network. Regulatory Norm No. 7 (NR-7), which deals with the Occupational Medical Control Program in Brazil, updated through Ordinance No. 567/2022, indicates that the notification of work-related injuries must be carried out immediately after the diagnosis or verification of the illness situation. There is no pre-determined period for making the notification, but the emphasis is that it be done immediately, in order to favor prevention and control measures in the work environment [23]. The Manual of Procedures for health services, on the other hand, indicates that the notification of the condition in SINAN must occur from the diagnosis of the disease and the establishment of a link with work [24].

The study had limitations that may be associated with the quality of secondary data. In this way, there is no control over the correct completion of the notifications included in the study, nor over the volume of underreporting, which may impact the results obtained. The period of the study is also a limiting factor, since the size of the disaster would require a longer follow-up time to show changes in the medium and long term. Due to the dimensions and repercussions of the disaster, the impact on workers’ mental health may be even greater than what was reported during the study period. In the case of mental health, it would be necessary to sensitize health units to register these disorders that manifest themselves over time. Therefore, medium and long-term monitoring of the injuries and diseases reported on SINAN is necessary for proper follow-up and planning of workers’ health actions.

## Conclusions

The study showed that there was an increase in the volume of notifications of work-related diseases and injuries, especially serious accidents, mental disorders and accidents involving biological material, after the collapse of the mining tailings dam in Brumadinho.

This increase suggests that after the disaster, worker health surveillance was intensified to record diseases arising from working conditions. On the other hand, there was an overload of the Unified Health System, which is responsible for treating work-related injuries and diseases.

It was shown that there were changes in the number of notifications between the periods before and after the mining disaster, indicating an impact on the health of workers that may persist over the years.

## Data Availability

The datasets used and/or analysed during the current study are available from the corresponding author upon reasonable request.
